# JOINING AND REMAINING: FACTORS THAT CONTRIBUTE TO MEMBERSHIP IN A VILLAGE

**DOI:** 10.1093/geroni/igz038.1028

**Published:** 2019-11-08

**Authors:** Ruth E Dunkle, Karen Harlow-Rosentraub, Garrett T Pace, Larry Coppard

**Affiliations:** University Of Michigan, Ann Arbor, Michigan, United States

## Abstract

Many older people want to age in place with one popular model of care being the Village Model. Understanding why people join and why they continue as members are important considerations for a Village to survive. Utilizing open-ended data from a representative sample of current members of a Village (N=100), we examined the reasons for people becoming members as well as continuing their membership. Twenty six percent of the sample were men, age ranged from 50-95, 30% lived alone and 58% believed that their health was very good. Data were coded by three researchers with discrepancies resolved by discussion to refine the codes. Three categories were identified: instrumental, social, and altruistic. The most frequent reason for joining was instrumental (35%) where the member wanted the services provided by the Village. It was also the most frequent reason for continuing membership (57%). An analysis was also conducted to examine predictors of reasons for joining and continuing membership in the Village. These included, age, gender, health, and living alone. Results indicate that men were less likely to join or continue their membership for instrumental reason compared to women, and members who live alone were more likely to become a member for social reasons. When age at entrance into the Village was examined, each increasing year of age was associated a .01 increase in the probability of continuing as a member for instrumental reasons. Findings provide guidance in issues related to sustaining membership in a Village.

